# Mitogen-activated protein kinase phosphatase-1 (MKP-1) impairs the response to anti-epidermal growth factor receptor (EGFR) antibody cetuximab in metastatic colorectal cancer patients

**DOI:** 10.1038/sj.bjc.6605612

**Published:** 2010-03-16

**Authors:** C Montagut, M Iglesias, M Arumi, B Bellosillo, M Gallen, A Martinez-Fernandez, L Martinez-Aviles, I Cañadas, A Dalmases, E Moragon, L Lema, S Serrano, A Rovira, F Rojo, J Bellmunt, J Albanell

**Affiliations:** 1 Medical Oncology Department, Hospital del Mar-IMAS, Barcelona 08003, Spain; 2 Cancer Research Program, IMIM-Hospital del Mar, Barcelona 08003, Spain; 3 Pathology Department, Hospital del Mar, Barcelona 08003, Spain; 4 Departament de Medicina, Universitat Autònoma de Barcelona, Bellaterra 08193, Spain; 5 Pathology Department, Fundación Jiménez Díaz, Madrid 28040, Spain; 6 Department of Medicine, Universitat Pompeu Fabra, Barcelona 08003, Spain

**Keywords:** MKP-1, cetuximab, CRC, RAS, molecular marker

## Abstract

**Background:**

The validation of KRAS mutations as a negative marker of response to anti-epidermal growth factor receptor (EGFR) antibodies has meant a seminal advance towards treatment individualisation of colorectal cancer (CRC) patients. However, as a KRAS wild-type status does not guarantee a response to anti-EGFR antibodies, a current challenge is the identification of other biomarkers of response. On the basis of pre-clinical evidence, we hypothesised that mitogen-activated protein kinase phosphatase-1 (MKP-1), a phosphatase that inactivates MAPKs, could be a mediator of resistance to anti-EGFR antibodies.

**Methods:**

Tumour specimens from 48 metastatic CRC patients treated with cetuximab-based chemotherapy were evaluated for KRAS and BRAF mutational status and MKP-1 expression as assessed by immunohistochemistry.

**Results:**

As expected, clinical benefit was confined to wild-type KRAS and BRAF patients. Mitogen-activated protein kinase phosphatase-1 was overexpressed in 16 patients (33%) and was not associated with patient baseline clinicopathological characteristics and KRAS mutational status. All patients with BRAF mutations (*n*=3) had MKP-1 overexpression. Among KRAS wild-type patients, MKP-1 overexpressors had a 7% response rate (RR), whereas patients not overexpressing MKP-1 had a 44% RR (*P*=0.03). Moreover, median time to progression was significantly longer in MKP-1 non-overexpressing patients (32 *vs* 13 weeks, *P*=0.009).

**Conclusion:**

These results support the concept of MKP-1 as a promising negative marker of response to cetuximab-based treatment in CRC patients with wild-type KRAS.

Epidermal growth factor receptor (EGFR) is a transmembrane tyrosine kinase receptor that, on ligand binding to its extracellular domain, is activated and autophosphorylated. This activates several intracellular signalling pathways that regulate crucial oncogenic properties, mainly the PI3K–PTEN–AKT and the mitogen-activated protein kinase (MAPK) RAS–RAF–MEK–ERK cascade (Hanahan and Weinberg, 2000; Albanell *et al*, 2001; Mendelsohn and Baselga, 2006). Moreover, EGFR inhibits the activation of the other two MAPKs, namely, p38 MAPK and c-Jun NH2-terminal kinase (JNK), which, contrary to ERK, drive apoptotic signals (Dhillon *et al*, 2007; Takeuchi and Ito, 2010). The unique position of EGFR as a regulator of several key oncogenic pathways, together with the fact that EGFR is frequently expressed in colorectal cancer (CRC), has made it an excellent therapeutic target for the treatment of CRC patients.

The approval of two anti-EGFR monoclonal antibodies (moAbs), cetuximab and panitumumab, for metastatic CRC (mCRC) has dramatically improved the outcome of these patients. Both cetuximab and panitumumab have shown response rates (RRs) of 10–15% as single agents (Cunningham *et al*, 2004; Van Cutsem *et al*, 2007) and ∼20% RR for cetuximab in combination with chemotherapy in unselected advanced mCRC patients (Cunningham *et al*, 2004). Moreover, anti-EGFR moAbs improve survival as salvage therapy in advanced mCRC (Jonker *et al*, 2007; Van Cutsem *et al*, 2007; Karapetis *et al*, 2008). The recent validation of KRAS mutations as a biomarker of negative response (or biomarker of resistance) to anti-EGFR therapies has meant a major revolution in the field of targeted therapies and in the treatment of mCRC patients (Amado *et al*, 2008; Karapetis *et al*, 2008; Bokemeyer *et al*, 2009; Van Cutsem *et al*, 2009). Activating mutations of the downstream EGFR protein KRAS are present in ∼40% of CRC patients. The most frequent KRAS mutations occur on codons 12 and 13, which impair ATPase activity, leading to a permanent activation of the KRAS–RAF–MEK–ERK pathway, despite EGFR blocking by cetuximab or panitumumab (Benvenuti *et al*, 2007; Di Fiore *et al*, 2007; De Roock *et al*, 2008; Lievre *et al*, 2008). After preliminary results in a retrospective cohort analysis (Lievre *et al*, 2008), the use of KRAS mutations as a marker of resistance to anti-EGFR moAbs was supported by a retrospective tumoural analysis of patients included in a randomised phase III clinical trial of panitumumab monotherapy *vs* best supportive care in chemotherapy-refractory mCRC patients. KRAS mutant patients had a statistically significant lower RR (0% *vs* 17%) and shorter progression-free survival (Amado *et al*, 2008). In the case of cetuximab, results of a retrospective analysis of KRAS mutational status in tumour biopsy samples of patients included in several randomised phase III clinical trials have confirmed that KRAS is a solid biomarker of resistance to cetuximab. The CO.17 clinical trial showed that clinical benefit to salvage cetuximab monotherapy was confined to wild-type patients (Karapetis *et al*, 2008). In the OPUS and CRYSTAL trials, the addition of cetuximab to conventional chemotherapy in first-line treatment for mCRC patients showed no clinical benefit in KRAS mutated patients, whereas KRAS wild-type patients had ∼60% of clinical responses and longer progression-free survival with the addition of cetuximab (Bokemeyer *et al*, 2009; Van Cutsem *et al*, 2009). Therefore, the Food and Drug Administration has recently restricted the indication for panitumumab and cetuximab to wild-type KRAS mCRC patients. However, wild-type KRAS does not guarantee response to anti-EGFR moAb, and a fraction of KRAS wild-type patients will receive cetuximab or panitumumab without deriving any benefit. Thus, it is now crucial to find other markers of response that will help us in selecting KRAS wild-type patients most likely to respond – or not respond – to anti-EGFR therapy.

Although other mutationally activated protein kinases downstream of EGFR are being evaluated as potential biomarkers of resistance to cetuximab, so far none of them has sufficient supporting evidence to be routinely used in clinical practice. The V600E BRAF mutation is present in ∼5–10% of CRC patients and is mutually exclusive with KRAS mutations. In a hypothesis-generating retrospective analysis of tumour biopsy samples of 113 patients treated with anti-EGFR moAb plus chemotherapy, BRAF-mutated patients did not respond to therapy and had a shorter progession-free survival and overall survival (OS) compared with BRAF wild-type patients (Di Nicolantonio *et al*, 2008). Nevertheless, these results have not been confirmed when retrospectively analysing the BRAF mutational status of a large phase III clinical trial that evaluated chemotherapy, anti-angiogenic therapy and cetuximab in first-line treatment of mCRC patients (Tol *et al*, 2009). The role of BRAF mutations as a prognostic factor, as well as its low prevalence, will limit the confirmation of BRAF as a marker of resistance to anti-EGFR therapies (Di Nicolantonio *et al*, 2008; Tol *et al*, 2009; Roth *et al*, 2010). Activating mutations of PI3K are present in ∼10–30% of mCRC patients and have also been suggested to be a negative biomarker of response to cetuximab, although there are contradictory results from different studies (Ogino *et al*, 2009; Prenen *et al*, 2009; Sartore-Bianchi *et al*, 2009). Upregulation of the PI3K–Akt axis by loss of PTEN is theoretically another marker of resistance to anti-EGFR moAb. Despite several positive retrospective series, the studies are limited by a lack of standardised PTEN immunohistochemistry scoring (Frattini *et al*, 2007; Loupakis *et al*, 2009; Sartore-Bianchi *et al*, 2009). Upregulation of EGFR ligands – amphiregulin and epiregulin – expression has also been shown to be a marker of resistance in an expression-array analysis (Khambata-Ford *et al*, 2007), and has been confirmed in a large retrospective study (Jacobs *et al*, 2009). TP53 mutations are another potential mechanism of resistance to anti-EGFR under evaluation (Oden-Gangloff *et al*, 2009).

Mitogen-activated protein kinase phosphatases (MKPs), also known as dual-specificity phosphatases, regulate the activity of MAPKs (ERK, JNK and p38) by dephosphorylating both threonine and tyrosine residues and therefore deactivating them (Wu, 2007; Keyse, 2008). The MKP family is composed of 12 members, of which MKP-1 (also referred to as dual-specificity phosphatase-1 and CL100) is the best characterised. MKP-1 is located in the nucleus and regulates the three MAPKs with different substrate preferences based on cell type and context (Chu *et al*, 1996; Keyse (2008)). MKP-1 is overexpressed in human tumours such as lung, breast and colon cancer, and has been involved in tumourigenesis (Loda *et al*, 1996; Vicent *et al*, 2004; Rojo *et al*, 2009). Oncogenic activation of MKP-1 is mediated by EGFR signalling by poorly understood mechanisms that include transcriptional regulation mediated by p38 and ERK (Xing *et al*, 1996; Li *et al*, 2001) and post-translational control by ERK (Brondello *et al*, 1999; Lin *et al*, 2003; Lin and Yang, 2006). Interestingly, our group and others are providing increasing evidence for a role of MKP-1 in acquisition of resistance to anti-cancer therapy. The molecular mechanism underlying MKP-1-mediated resistance to anti-cancer drugs is in part due to the activation of JNK-driven apoptosis by several anti-tumour agents such as anthracyclines, taxanes, cisplatin, proteasome inhibitors and more recently anti-EGFR drugs. High levels of MKP-1 inhibit JNK and counterbalance the cytotoxic effects of such drugs (Sanchez-Perez *et al*, 1998, 2000; Small *et al*, 2004, 2007; Wang *et al*, 2006, 2007; Workman and de Bono, 2008; Rojo *et al*, 2009). With respect to anti-EGFR therapy, *in vitro* results with the anti-EGFR drug AG1478 showed that MKP-1-modulated JNK activation was critical for drug-induced apoptosis. Moreover, ectopic expression of MKP-1 suppressed JNK-mediated AG1417 apoptosis, leading to resistance to anti-EGFR therapy (Takeuchi *et al*, 2009). Thus, MKP-1 overexpression is a potential negative biomarker of response to certain anti-cancer agents including anti-EGFR therapy. Furthermore, preliminary pre-clinical work from our group found an association between high MKP-1 expression and resistance to cetuximab in CRC cell lines with wild-type KRAS. MKP-1 expression was markedly lower in DiFi cells (sensitive to cetuximab) compared with SW48 cells (resistant to cetuximab) (A Dalmases and C Montagut, unpublished data).

On the basis of the extensive evidence of the implication of MKP-1 in resistance to anti-cancer agents, as well as on the preliminary pre-clinical evidence by our group and others of the implication of MKP-1 in anti-EGFR therapy resistance (Takeuchi *et al*, 2009), we hypothesised that MKP-1 expression in human CRC tumours may be a marker of resistance to anti-EGFR moAb. We therefore analysed the impact of MKP-1 on clinical outcome in mCRC patients treated with cetuximab, with a special interest in KRAS wild-type patients.

## Materials and methods

### Patient characteristics and clinical evaluation

We retrospectively selected 48 consecutive patients with histologically confirmed mCRC treated with cetuximab-based chemotherapy at Hospital del Mar between 2004 and 2009. Patients’ selection criteria were based on tumour tissue availability for molecular analysis. Cetuximab was administered as a loading dose of 400 mg m^−2^, followed by 250 mg m^−2^ every week intravenously. Clinical data and follow-up were obtained from the patients’ medical records. Tumour response was evaluated retrospectively according to the response evaluation criteria in solid tumours (Therasse *et al*, 2000). Patients with complete response, partial response or stable disease were considered to have controlled disease (CD) (Oden-Gangloff *et al*, 2009). This study was approved by the Ethics Board of the Hospital and was performed according to Institutional Guidelines.

### Mutational analyses

DNA was extracted from two 15-μm sections of paraffin-embedded tissue using a QIAamp Tissue Kit (QIAGEN GmbH, Hilden, Germany) according to the manufacturer's protocol. In those cases in which <50% of tumoural cells were present in the sample, manual microdissection of tumoural tissue was performed. Primers for KRAS (codons 12 and 13) and BRAF V600E amplification were designed using Primer Express software (Applied Biosystems, Foster City, CA, USA) using NG_007524.1 (KRAS) and NG_007873.1 (BRAF) sequences and were as follows: KRAS-F: 5′-TTACGATACACGTCTGCAGTCAAC-3′ KRAS-R: 5′-AAAGAATGGTCCTGCACCAGTAATA-3′ BRAF-F: 5′-CGGCTCCTAAAGCAATGGC-3′ BRAF-R: 5′-CAGCATCTCAGGGCCAAAAA-3′. DNA amplification was performed by PCR under the following conditions: initial denaturation for 10 min at 95 °C, 40 cycles consisting of: 1 min at 95 °C, 1 min at 54 °C (KRAS) or at 55 °C (BRAF) and 1 min at 72 °C, and a final step at 72 °C for 10 min. Mutation analysis was performed by direct sequencing with BigDye v3.1 (Applied Biosystems) according to the manufacturer's instructions and analysed on an ABI3730XLSequencer (Applied Biosystems).

### Immunohistochemistry

Immunohistochemistry was performed according to the methodology previously described by our group (Rojo *et al*, 2009). In brief, formalin-fixed paraffin-embedded 3 μm tissue sections were used for immunostaining using the Dako-Link platform. After deparaffinisation in xylene and graded alcohols, heat antigen retrieval was carried out in pH9 EDTA-based buffer (Dako, Carpinteria, CA, USA). Endogenous peroxidase was blocked by immersing the sections in 0.03% hydrogen peroxide for 5 min. Slides were incubated with anti-MKP-1 primary antibody for 1 h at room temperature, followed by incubation with the appropriate anti-Ig horseradish peroxidase-conjugated EnVision polymer (Dako) to detect antigen–antibody complexes. Sections were then visualised with 3,3′-diaminobezidine as chromogen and counterstained with haematoxylin. The specificity of the staining with anti-MKP-1 antibody was controlled by pre-incubating the antibody with antigen (blocking peptide), and performing immunostaining of tissue sections and immunoblotting of BT-474 breast cancer cell extracts. In addition to human specimens, renal tissues were obtained from wild-type and MKP-1 gene knockout mice, generously provided by Bristol-Myers Squibb Co. (Princeton, NJ, USA), and used for validation of MKP-1 assay. These mouse samples were processed with the same reagents and procedures as used for human samples.

Immunohistochemical evaluation was carried out by three independent observers (MI, MA and FR). To score a cell as positive, nuclear staining was required for MKP-1 expression. The expression was evaluated by calculating a semiquantitative histoscore (H-score) that included the determination of both the percentage of stained target cells and staining intensity (low, medium or high), as described in Rojo *et al*, (2009). The final score was determined after applying a weighting factor to each estimate and the following formula was used: H-score=(low%) × 1+(medium%) × 2+(high%) × 3; the results ranged from 0 to 300. MKP-1 was expressed in histologically normal colon epithelial cells and the pattern and intensity of staining were similar in all assayed specimens. MKP-1 overexpression was considered when the intensity of staining in the nuclei of tumour cells was higher than that observed in corresponding normal epithelial cells (Rojo *et al*, 2009).

### Statistical analysis

Fisher's exact test was used to evaluate the association between MKP-1 expression with dichotomous clinical and molecular variables. Response to cetuximab-based therapy (responders *vs* non-responders) according to KRAS or BRAF mutational status or MKP-1 or EGFR expression was assessed by Fisher's exact test. The time to progression (TTP) was defined as the time from the start of cetuximab-based treatment until documented tumour progression or death. The Kaplan–Meier method was used to estimate TTP and OS and the log-rank test to compare survival curves. All statistical tests were conducted at the two-sided 0.05 level of significance. Statistical analysis was performed with SPSS Statistical Software, 17.0 version (SPSS, Inc., Chicago, IL, USA).

## Results

### Patient baseline characteristics and clinical response to cetuximab

A total of 48 patients with mCRC treated with cetuximab-based chemotherapy were included in this study. Of them, 47 had been previously treated with chemotherapy, most of them (83%) had previously received two or more lines of salvage treatment. Administration of cetuximab was combined with irinotecan in 92% of the patients. Evaluation of response to cetuximab based-therapy showed that 11 patients responded to treatment (11 partial responses; 0 complete responses) with a median TTP of 27 weeks (range 1–66 weeks). Non-responders (stable disease in 15 patients; progression disease in 22 patients) had a median TTP of 13 weeks (range 4–65 weeks). Patient baseline characteristics are shown on Table 1.

### KRAS mutational status and clinical response to cetuximab

**Figure 1:**
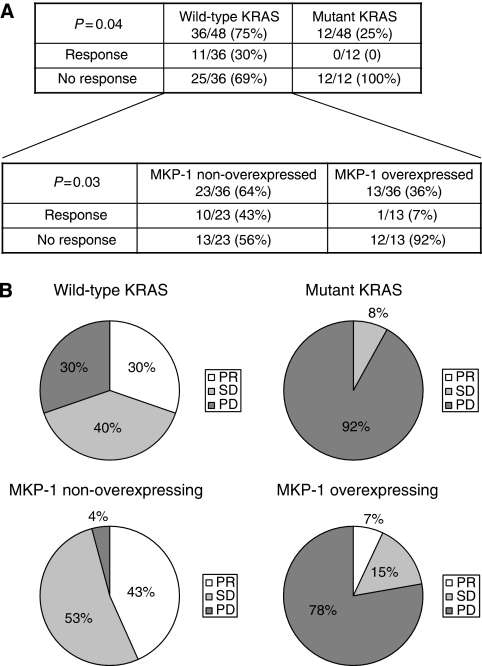
KRAS mutations correlate with a lack of response to cetuximab. In KRAS wild-type patients, mitogen-activated protein kinase phosphatase-1 (MKP-1) overexpression is inversely correlated with response to cetuximab. (**A**) The number (and percentage) of patients with response and non-response (stable disease (SD)+progressive disease (PD)) to cetuximab are indicated according to KRAS mutational status and MKP-1 expression. (**B**) Pie charts showing the percentage of patients showing partial response (PR), SD and PD according to KRAS mutational status and MKP-1 expression.

The mutational status of KRAS was assessed in all 48 patients included in the study. KRAS mutations were found in 12 patients (25%). Such a low percentage compared with that in previous reports is probably due to the fact that after Health Authorities approval to limit the use of anti-EGFR moAb to KRAS wild-type mCRC patients, KRAS mutant cases have not received cetuximab-based therapy at our Institution. KRAS mutations were as follows: G13D in five patients; G12D in three patients; G12V in three patients; and G12A in one patient. In all, 11 out of 36 KRAS wild-type patients (30%) responded to cetuximab, whereas none of the 12 patients (0%) harbouring a KRAS mutation had a partial response (*P*=0.04). We also measured CD, as recently reported (Oden-Gangloff *et al*, 2009). KRAS wild-type patients showed statistically significant improvement in CD compared with mutant KRAS patients (69% *vs* 8%, respectively). The median TTP for KRAS wild-type patients was 25 weeks *vs* 8 weeks for KRAS mutant patients (*P*=0.01). Patients carrying KRAS-mutated tumours tended to have shorter OS, although the difference did not reach statistical significance (*P*=0.1). Taken together, such findings confirm that KRAS mutations inversely correlate with clinical benefit from cetuximab therapy (Figure 1).

### BRAF mutational status and clinical response to cetuximab

Three patients (6%) harboured BRAF mutations, which were mutually exclusive with the presence of KRAS mutations. None of the three patients with BRAF mutations responded to cetuximab compared with 33% responses to cetuximab in BRAF wild-type patients. This difference was not statistically significant, probably because of the low incidence of BRAF (*P*=0.54). Median TTP was higher in wild-type BRAF patients than in mutant BRAF patients (25 *vs* 7 weeks), although this correlation did not reach statistical significance (*P*=0.53).

### MKP-1 expression and correlation with clinical and molecular characteristics

**Figure 2:**
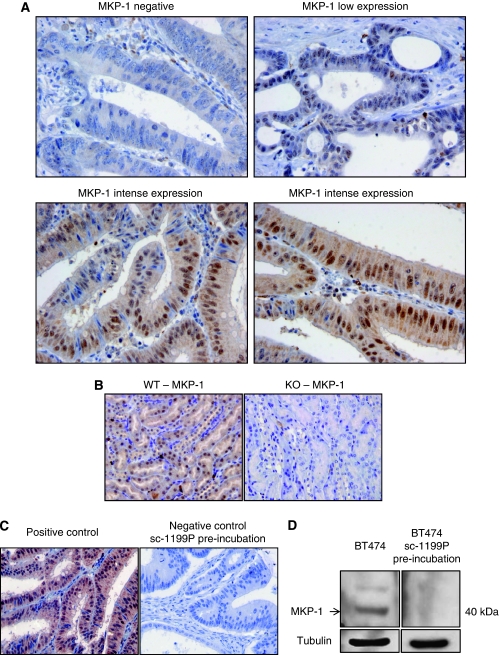
Mitogen-activated protein kinase phosphatase-1 (MKP-1) expression in colon cancer. (**A**) Representative colon adenocarcinoma specimens showing undetected (negative) MKP-1 expression, low expression and intense diffuse staining in tumour cells. Malignant cells expressed MKP-1 nuclear staining; mild cytoplasmic staining is occasionally noted. Stroma cells (fibroblast and endothelial cells) also showed a low level of MKP-1 expression, which is not detected in lymphocytes. (**B**) The specificity of immunostaining was probed by assaying renal tissue sections obtained from wild-type and MKP-1 gene knockout mice. Specimens were processed using the same reagents and procedures as used in human samples. No staining was observed in knockout tissue and intense nuclear expression was detected in renal tubes. (**C**) Positive and negative controls. The same tumour specimen considered as positive control was assayed by pre-incubation of primary antibody with a specific blocking peptide, showing no staining. (**D**) MKP-1 expression explored by western blot from BT-474 total lysates, using the same primary antibody and pre-incubated with a blocking peptide. The molecular size of MKP-1 was ∼40 kD. The protein was not detected under blocking peptide pre-incubation.

Activated MKP-1 (i.e., nuclear staining), as assessed by immunohistochemistry, was overexpressed in 16 patients (33% Table 1). Occasional and faint cytoplasmic MKP-1 was noted in malignant cells. Histologically normal epithelial cells exhibited weak and diffuse MKP-1 staining in the nuclei. A low level of MKP-1 expression was also present in stroma cells (fibroblast and endothelial cells) but it was not detected in lymphocytes (Figure 2).

No significant correlation was found between MKP-1 expression and clinical characteristics of patients, including age (<65 *vs* ⩾65 years old), sex, tumour primary site (colon *vs* rectum), tumour size (T1–2 *vs* T3–4), nodal status (positive *vs* negative), cetuximab regimen (irinotecan *vs* oxaliplatin), number of previously received chemotherapy metastatic lines (<2 *vs* ⩾2 lines), hepatic, lung, ascites and other metastases (present *vs* absent for each metastatic site) and metastasectomy. MKP-1 expression was not linked to expression of EGFR as assessed by immunohistochemistry (*P*=0.61). The expression of MKP-1 was not correlated with KRAS mutational status as shown by 19% of MKP-1 overexpressors harbouring a KRAS mutation *vs* 28% of MKP-1 non-overexpressors with mutant KRAS (*P*=0.72). It is worth noting that all three patients with BRAF mutations had MKP-1 overexpression (*P*=0.04) (Table 1).

### MKP-1 expression and clinical response to cetuximab-based treatment in all patients

Only 1 of 16 patients with MKP-1 overexpression (6%) had a partial response to treatment, whereas 10 of 32 non-overexpressing MKP-1 patients (31%) responded to cetuximab, although the correlation was not statistically significant (*P*=0.074). Moreover, CD was achieved in 19% of patients with MKP-1 overexpression, compared with 72% of patients with low MKP-1 protein levels (*P*=0.001). The median TTP was lower in MKP-1 overexpressing patients, compared with patients with non-overexpressed MKP-1 (13 *vs* 27 weeks; *P*=0.43). No statistically significant differences were observed by OS analysis (*P*=0.5).

### MKP-1 expression and clinical response to cetuximab in KRAS wild-type patients

**Figure 3:**
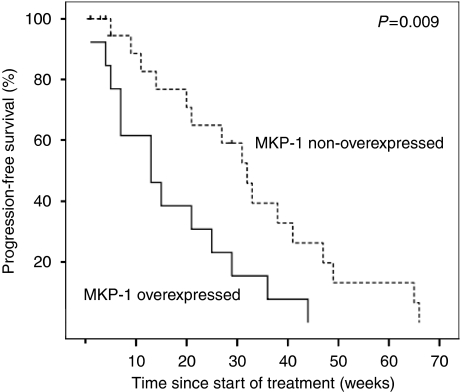
Patients with mitogen-activated protein kinase phosphatase-1 (MKP-1) overexpression show shorter median time to progression (TTP) than MKP-1 non-overexpressing patients in KRAS wild-type colon cancer patients treated with cetuximab (13 *vs* 32 weeks, *P*=0.009).

To address the clinically relevant need to identify KRAS wild-type patients who may respond to cetuximab, we analysed MKP-1 expression in the subset of KRAS wild-type patients. Among KRAS wild-type patients, only 1 out of 13 MKP-1 overexpressing patients (7%) responded to cetuximab, whereas 10 out of 23 patients (43%) with non-overexpressed MKP-1 responded to cetuximab (*P*=0.03, Figure 1). Furthermore, wild-type KRAS patients with low MKP-1 levels had 96% CD as compared with 22% CD in wild-type KRAS patients with high MKP-1 levels (*P*<0.0001, Figure 1). Patients with wild-type KRAS and overexpressed MKP-1 had a shorter median TTP than wild-type KRAS patients with non-overexpressed MKP-1 (13 *vs* 32 weeks, *P*=0.009, Figure 3). KRAS wild-type individuals with non-overexpressed MKP-1 showed a trend towards longer OS, which was not statistically significant (*P*=0.1). Such results suggest MKP-1 as a potential predictive factor of failure to respond to cetuximab in KRAS wild-type patients.

### MKP-1 expression and clinical response to cetuximab in KRAS and BRAF wild-type patients

Given the increasing amount of evidence on a role for BRAF as a marker of resistance to anti-EGFR moAb and to exclude a possible bias, the predictive role of MKP-1 to cetuximab-based therapy was assessed in KRAS and BRAF wild-type patients. Among KRAS/BRAF wild-type patients, only 1 out of 10 patients (10%) with overexpressed MKP-1 responded to cetuximab, compared with 10 out of 23 patients (43%) with non-overexpressed MKP-1 (*P*=0.1). It is of importance that CD was only observed in two patients with high MKP-1 expression levels, whereas 22 of the 23 patients with MKP-1 low expression had CD (*P*=0.0001). Among KRAS and BRAF wild-type patients, those with MKP-1 overexpression had shorter TTP than patients with low MKP-1 levels (32 *vs* 13 weeks, *P*=0.006).

## Discussion

This study suggests MKP-1 as a novel and promising biomarker of response to cetuximab in mCRC patients, particularly relevant to certain KRAS wild-type patients who will not benefit from cetuximab therapy.

The selection of patients to be treated with targeted therapies based on useful and validated biomarkers is crucial to maximise clinical efficacy while minimising toxicities and optimising the use of currently constrained financial resources (Baselga, 2006; McDermott *et al*, 2007; Workman and de Bono, 2008). The validation of KRAS mutations as a negative biomarker of response to anti-EGFR therapies such as cetuximab has meant a major revolution in the treatment of CRC patients. The number of KRAS mutations in this study (25%) was lower than that previously reported, partly because of the fact that after June 2008, mutant KRAS patients were not treated with cetuximab at our Institution according to the results presented at the 44th ASCO Annual Meeting (Bokemeyer *et al*, 2009; Van Cutsem *et al*, 2009). As expected, in our series, clinical response was confined to wild-type KRAS patients, who also had a statistically significantly longer TTP than mutant KRAS patients.

In this study, BRAF mutations correlated with resistance to cetuximab-based therapy, although the decrease in median TTP was not statistically significant. We cannot rule out that this may be related to an insufficient sample size to detect differences in such a low-frequency occurring event. The response data confirm the results of previous studies that showed that BRAF mutations were linked to cetuximab efficacy in mCRC patients (Di Nicolantonio *et al*, 2008). Nevertheless, recent results in mCRC patients treated with chemotherapy, bevacizumab and cetuximab as first-line therapy showed that BRAF mutations had a role as a prognostic factor but not as a predictive marker of response to cetuximab (Tol *et al*, 2009). Moreover, BRAF mutations have prognostic value in stage II and III CRC patients (Roth *et al*, 2010). Such apparently contradictory results may in part be explained by the possible acquisition of BRAF mutations in advanced stages of the disease and suggest that the role of BRAF as a predictive marker of resistance to cetuximab might be restricted to patients previously treated with chemotherapy. In this sudy, 83% of patients had received two or more previous lines of chemotherapy, and only one patient was treated with cetuximab as first-line therapy for mCRC. Another controversial marker of anti-EGFR moAb efficacy is the target itself. EGFR protein, as assessed by immunohistochemistry, was overexpressed in 76% of tumours (data not shown) and did not correlate with response to cetuximab as previously demonstrated in other studies (Cunningham *et al*, 2004; Saltz *et al*, 2004; Chung *et al*, 2005).

Our data showed that overexpression of MKP-1, as assessed by immunohistochemistry, was correlated with resistance to cetuximab-based chemotherapy in mCRC patients, and suggests a role for MKP-1 as a negative predictive biomarker of response to cetuximab, particularly in KRAS wild-type patients. Although this study is the first to correlate MKP-1 levels to cetuximab resistance in CRC patients, the implication of other MKPs in modulating response to anti-EGFR therapy has previously been suggested. A gene expression profile analysis identified high MKP-2 levels (also known as dual-specificity phosphatase-4) in cetuximab-resistant patients (Khambata-Ford *et al*, 2007). Moreover, an elegant abstract presented at the 2009 ASCO Annual Meeting showed that MKP-2 expression levels as determined by quantitative reverse transcriptase PCR identifies a subgroup of patients with shorter median OS to cetuximab therapy among KRAS wild-type mCRC patients (De Roock *et al*, 2009).

As all patients included in this study received cetuximab in combination with chemotherapy (irinotecan in 92% of the patients), it cannot be excluded that MKP-1-based clinical outcome is influenced, at least in part, by its interaction with chemotherapy. Although so far there is no reported evidence of a role of MKP-1 in conferring resistance to irinotecan, it will certainly be necessary to conduct randomised clinical trials and extensive pre-clinical modelling to exclude such hypotheses. A recently published paper reports that other cetuximab predictive markers such as KRAS and BRAF mutations do not preclude benefit from irinotecan or oxaliplatin chemotherapy (Richman *et al*, 2009).

Molecular mechanisms underlying MKP-1-mediated resistance to cetuximab are poorly understood. A possible explanation is inhibition of JNK-mediated apoptosis by high MKP-1 levels, as has recently been reported by Takeuchi *et al* (2009). On the other hand, a recently published interesting hypothesis-generating study supports p53 mutations as a potential marker of response to cetuximab (Oden-Gangloff *et al*, 2009). MKP-1 has been shown to be transcriptionally regulated by p53, and mutations of p53 abrogate p53-dependent transcription of MKP-1 *in vitro* (Yang and Wu, 2004; Liu *et al*, 2008). Thus, it could be speculated that the association between p53 mutations and better clinical outcome in cetuximab-treated patients is in part explained by a decrease in the expression of MKP-1, although this molecular association needs to be further characterised.

Interestingly, mutant KRAS tumours have been shown to express high constitutive levels of MKP-1, MKP-2 and MKP-3, probably as part of the regulatory feedback loop to attenuate the high activation of ERK by mutant KRAS (Bild *et al*, 2006). Moreover, functional studies in a KRAS mutant CRC murine model has confirmed MKP-3 high levels, and high MKP-2 and MKP-3 expressions have been described in human tumour biopsy samples from mutant KRAS CRC patients (Haigis *et al*, 2008; De Roock *et al*, 2009). However, in this study, we found that MKP-1 basal levels were not linked to KRAS mutations. It is worth noting that the presence of BRAF V600E mutations was associated with MKP-1 overexpression in all the cases, although the number of patients was insufficient to achieve a significant correlation.

Collectively, our results suggest a role for MKP-1 in predicting failure to respond to cetuximab-based chemotherapy in KRAS wild-type CRC patients.

## Figures and Tables

**Table 1:** Patient baseline characteristics and clinical response by MKP-1 status

**Characteristics (number of patients)**	**Number (%) overexpressing MKP-1**	***P*-value**
Number of patients (*n*=48)	16 (33)	
		
*Age (years)*		
<65 (*n*=26)	7 (27)	NS
⩾65 (*n*=22)	9 (41)	
		
*Sex*		
Male (*n*=31)	12 (39)	NS
Female (*n*=17)	4 (23)	
		
*Site of primary tumour*		
Colon (*n*=36)	11 (30)	NS
Rectum (*n*=12)	5 (42)	
		
*Tumour size*		
T1–T2 (*n*=2)	0 (0)	NS
T3–T4 (*n*=46)	16 (35)	
		
*Nodal status*		
Negative (*n*=4)	2 (50)	NS
Positive (*n*=44)	14 (32)	
		
*Cetuximab regimen*		
Irinotecan based (*n*=44)	15 (34)	NS
Oxaliplatin based (*n*=4)	1 (25)	
		
*Number of previous chemotherapy*		
<2 (*n*=8)	3 (37)	NS
⩾2 (*n*=40)	13 (32)	
		
*Sites of metastasis*		
Hepatic (*n*=33)	13 (39)	NS
Lung (*n*=21)	7 (33)	NS
Peritoneal (*n*=13)	5 (38)	NS
Other (*n*=16)	4 (25)	NS
		
*Metastasectomy*		
Yes (*n*=6)	3 (50)	NS
No (*n*=42)	13 (31)	
		
*Mutational status*		
KRAS mutation (*n*=12)	3 (25)	NS
BRAF mutation (*n*=3)	3 (100)	0.04
		
*Clinical response*		
Partial response (*n*=11)	1 (9)	
Stable disease (*n*=15)	2 (13)	
Progression disease (*n*=22)	13 (59 )	

Abbreviations: MKP-1=mitogen-activated protein kinase phosphatase-1; NS=not significant.
